# Strain-specific quantification of root colonization by plant growth promoting rhizobacteria *Bacillus firmus* I-1582 and *Bacillus amyloliquefaciens* QST713 in non-sterile soil and field conditions

**DOI:** 10.1371/journal.pone.0193119

**Published:** 2018-02-15

**Authors:** Hajeewaka C. Mendis, Varghese P. Thomas, Patrick Schwientek, Rauf Salamzade, Jung-Ting Chien, Pramuditha Waidyarathne, Joseph Kloepper, Leonardo De La Fuente

**Affiliations:** 1 Department of Entomology and Plant Pathology, Auburn University, Auburn, AL, United States of America; 2 Crop Science Division, Biologics, Bayer, West Sacramento, CA, United States of America; 3 Centre for Advanced Computational Solutions, Lincoln University, Christchurch, New Zealand; Karnatak University, INDIA

## Abstract

*Bacillus amyloliquefaciens* QST713 and *B*. *firmus* I-1582 are bacterial strains which are used as active ingredients of commercially-available soil application and seed treatment products Serenade^®^ and VOTiVO^®^, respectively. These bacteria colonize plant roots promoting plant growth and offering protection against pathogens/pests. The objective of this study was to develop a qPCR protocol to quantitate the dynamics of root colonization by these two strains under field conditions. Primers and TaqMan^®^ probes were designed based on genome comparisons of the two strains with publicly-available and unpublished bacterial genomes of the same species. An optimized qPCR protocol was developed to quantify bacterial colonization of corn roots after seed treatment. Treated corn seeds were planted in non-sterile soil in the greenhouse and grown for 28 days. Specific detection of bacteria was quantified weekly, and showed stable colonization between ~104–10^5^ CFU/g during the experimental period for both bacteria, and the protocol detected as low as 10^3^ CFU/g bacteria on roots. In a separate experiment, streptomycin-resistant QST713 and rifampicin-resistant I-1582 strains were used to compare dilution-plating on TSA with the newly developed qPCR method. Results also indicated that the presence of natural microflora and another inoculated strain does not affect root colonization of either one of these strains. The same qPCR protocol was used to quantitate root colonization by QST713 and I-1582 in two corn and two soybean varieties grown in the field. Both bacteria were quantitated up to two weeks after seeds were planted in the field and there were no significant differences in root colonization in either bacteria strain among varieties. Results presented here confirm that the developed qPCR protocol can be successfully used to understand dynamics of root colonization by these bacteria in plants growing in growth chamber, greenhouse and the field.

## Introduction

Plant growth-promoting rhizobacteria (PGPR) are a group of bacteria that colonize plant roots and provide beneficial effects on plant growth and development [1]. Many diverse bacterial genera such as *Alcaligenes*, *Arthrobacter*, *Azoarcus*, *Azospirillum*, *Azotobacter*, *Bacillus*, *Burkholderia*, *Clostridium*, *Enterobacter*, *Gluconacetobacter*, *Klebsiella*, *Pseudomonas*, *and Serratia* include specific strains which are reported as PGPR [2–5]. PGPR have direct or indirect effects on plant growth promotion and improved crop yield. Direct effects of PGPR include providing plants with fixed nitrogen and phytohormones, increasing the availability of nitrogen, soluble phosphate and minerals in the soil and control or inhibition of the activity of plant pathogens [6–9]. Some PGPR are also responsible for promoting plant growth indirectly by eliciting Induced Systemic Resistance (ISR) [10–13].

Due to an increased public concern on the use of chemicals in agriculture [5], there has been a growing demand for PGPR as an alternative to synthetic fertilizer and chemical pesticides [14–16]. Use of PGPR in agriculture provides many benefits. PGPR have been used as a component of an integrated management system to reduce or to replace the use of chemical fertilizer and pesticides [3]. Members of the genus *Bacillus* produce heat and desiccation-resistant spores and are more amenable to commercial formulation. Therefore, most of the commercially available PGPR products contain *Bacillus* strains [10]. Many of these *Bacillus* spp. are developed as biocontrol agents of plant pests [5]. *Bacillus* species such as *B*. *amyloliquefaciens*, *B*. *licheniformis*, *B*. *pumilus* and *B*. *subtilis* are available in the market as biofungicide formulations [17].

*Bacillus amyloliquefaciens* QST713 (synonymous with *Bacillus subtilis* QST713) and *B*. *firmus* I-1582 are two key bacterial strains which are active ingredients in commercially-available biological products Serenade^®^ and VOTiVO^®^, respectively These bacteria colonize plant roots, promoting plant growth and offering protection against pathogens. VOTiVO^®^ is commercially available as a seed treatment to provide protection against nematode infection [18]. In addition, *B*. *firmus* I-1582 is also the active ingredient in Nortica^®^ in turf grasses. *B*. *firmus* strains reduce populations of *Meloidogyne incognita* in tomato roots and *Radopholus similis*, *Ditylenchus dipsaci* and *Heterodera glycines in vitro* [19,20]. Serenade^®^ is recommended as a soil treatment for managing soilborne and seedling diseases. *B*. *amyloliquefaciens* strains have shown antifungal activity against *Fusarium oxysporum* [21], *Puccinia striiformis* [22], *Rhizoctonia solani* [23], and *Pythium aphanidermatum* [24].

The term “root colonization” of PGPR describes the active growth of introduced bacteria on or around roots in the presence of indigenous microflora [1]. Research on PGPR as biocontrol agents shows that root colonization is a prerequisite for PGPR activity [25,26]. Therefore, quantification of root colonization by PGPR is an essential part of discovering and evaluating promising PGPR strains. Dilution-plate counting is a common method used in quantification of root colonizing bacteria. This method can be used successfully in gnotobiotic systems under sterile conditions [27]. However, dilution-plate counting could underestimate the number of bacteria associated with roots due to some of the cells failing to grow into colonies under the plating conditions [28]. The use of dilution-plating without a selective media is impracticable in the presence of native microflora. Furthermore, dilution-plating could be laborious and sample processing has to be carried out immediately after sampling. Therefore, a faster and more convenient quantification method should be able to differentiate the inoculated strain from indigenous rhizosphere bacterial community. Since standard microscopic methods and dilution-plate counting-based methods heavily rely on morphological characteristics of the bacteria, these methods are of limited value in quantification of PGPR root colonization where morphology is indistinguishable from other common microflora [1,27,28]. However, PGPR strains carrying antibiotic resistance determinants can also be used in dilution-plate method in the presence of native microflora. The use of PGPR strains genetically engineered to express fluorescent proteins is helpful in studying root colonization by PGPR under sterile conditions [29,30]. However, this method is of limited use under non-sterile conditions in greenhouse or in field, and for the use on wild-type strains.

The objective of this study was to develop a strain-specific molecular technique based on qPCR to detect and quantify root colonization of two PGPR strains *B*. *amyloliquefaciens* QST713 and *B*. *firmus* I-1582. We were able to quantifiy root colonization of *B*. *amyloliquefaciens* QST713 and *B*. *firmus* I-1582 strains in sterile soil, non-sterile soil in the greenhouse and non-sterile soil in the field.

## Materials and methods

### Bacterial strains and growth conditions

*Bacillus amyloliquefaciens* QST713 was previously described as *Bacillus subtilis* QST713. At the time of filing U.S. Patent Application No. 09/074,870 in 1998, the strain was designated as *B*. *subtilis* based on classical, physiological, biochemical and morphological methods. Taxonomy of the *Bacillus* species has evolved since then, especially in light of advances in genetics and sequencing technologies, such that species designation is based largely on DNA sequence rather than the methods used in 1998. Bayer is currently in the process of revising the *B*. *subtilis* designation of QST713, changing it to *B*. *amyloliquefaciens*, as would be expected currently based solely on genome sequence comparison and inferred taxonomy. For concordance with current taxonomic classification, the bacterial isolate formerly described as *B*. *subtilis* QST713 will be referred here as *B*. *amyloliquefaciens* QST713. *B*. *firmus* I-1582 and *B*. *amyloliquefaciens* QST713 were grown at 28°C with shaking at 150 rpm in Tryptic soy broth (TSB, pH 8.0) (BD Diagnostic Systems, Sparks, MD) from frozen glycerol stocks. Streptomycin (Sm)-resistant *B*. *amyloliquefaciens* QST713 (*B*. *amyloliquefaciens* QST713 –Sm) and rifampicin-resistant (Rif) *B*. *firmus* I-1582 (*B*. *firmus* I-1582-Rif) strains were grown in TSB or Tryptic soy agar (TSA) plates with streptomycin at 100 μg/ml (TSA_Sm_) or rifampicin at 100 μg/ml (TSA_Rif_). Spores of all *Bacillus* strains used in this study were produced by streaking 100 μL of two-day-old liquid cultures in sporulation media plates containing 3.3 g of Proteose peptone No. 3 (Becton, Dickinson and Company, Sparks, MD), 1.0 g of Beef extract powder (Neogen Corporation, Lansing, MI), 5.0 g of NaCl, 2.0 g of K_2_PO_4_, 1.0 g of KCl, 0.25 g of MgSO_4_, 0.01 g of MnSO_4_, 5.0 g of Lactose, 18.0 g of Agar (Becton, Dickinson and Company, Sparks, MD) in 1 L of MiliQ water. The sporulation plates were incubated for 2 weeks at 28^°^C.

### Plant inoculation with bacterial treatments

#### Seed treatment

A spore suspension of *B*. *amyloliquefaciens* QST713 or *B*. *firmus* I-1582 was obtained by adding sterile MiliQ water into several sporulation plates made as described above. The number of spores in the spore suspension was determined after heating the spore suspension at 80°C for 20 minutes in a water bath followed by dilution-plating. The spore suspension was then centrifuged at 13,000 rpm for 10 minutes to prepare a spore suspension with 2.5x10^8^ spores in 125 μl. This spore suspension and 25 corn seeds were added to a ziplock bag. A bubble was formed at the bottom of the ziplock bag by twisting the top of the bag towards the bottom. The bag is then shaken to distribute spore suspension onto corn seeds. This step was repeated until there was no visible liquid droplets in the bag. Bags were kept open in the laminar flow hood to air dry (Breeanne Schillianskey, personal communication).

#### Growth chamber

Corn seeds (2H723 Mycogen, Dow AgroSciences LLC, Indianapolis, IN) were treated with 10^7^, 10^5^ and 10^3^ spores of *B*. *firmus* I-1582; or 10^6^, 10^4^ and 10^2^ spores of *B*. *amyloliquefaciens* QST713 strains. Seeds were placed on sterile sand in 50 ml centrifuge tubes covered with aluminum foil and 1 ml of sterile water with corresponding amount of spores were drenched onto the seeds. Seeds were then covered with sterile sand and placed in growth chamber for 3 weeks. Two corn seedlings from each treatment were removed at 14 and 21 days after planting. Seedlings were watered once in every three days and temperature and relative humidity was maintained at 25^o^ C and 75% in the growth chamber.

#### Greenhouse

Corn seeds (2H723 Mycogen, Dow AgroSciences LLC, Indianapolis, IN) were treated with 10^7^ spores of *B*. *firmus* I-1582 and *B*. *amyloliquefaciens* QST713 strains separately, and 10^7^ spores of each strain together. Corn seeds were also treated with *B*. *firmus* I-1582-Rif and *B*. *amyloliquefaciens* QST713-Sm as described above. Seeds were then planted in sandy loam soil (pH = 7.4) in conetainers and grown in the greenhouse for 4 weeks. Three corn seedlings from each treatment were removed from the conetainer at 3, 7, 14, 21 and 28 days after planting.

#### Field

Seeds from two commercial maize and soybean hybrids with herbicide tolerance traits were treated with 10^6^ spores of either *B*. *firmus* I-1582 or *B*. *amyloliquefaciens* QST713 and grown in research fields of Crop Science Division, Bayer in Tifton, GA and Champaign, IL. Soybean and corn seedlings were carefully uprooted at 2 weeks after planting and shipped under cold storage to the De La Fuente lab at Auburn University. Root samples were processed and stored at -80^o^ C in 50 ml centrifuge tubes until beadbeating and DNA extraction (see below).

### DNA extraction from plant roots and in vitro cultures

Seedlings were removed from conetainers or conical tubes and shaken gently to remove loosely-attached soil from the roots. Approximately 5 g of roots were cut into 2–3 cm-long pieces and collected in a 7 ml plastic vial (BioSpec Products Inc, Bartlesville, OK) with 1.5–2 g of 2 mm Zirconia beads (BioSpec Products Inc, Bartlesville, OK). An aliquot of 2 ml of sterile water was added to each vial containing roots and Zirconia beads, secured in a Mini-BeadBeater-96 (BioSpec Products Inc, Bartlesville, OK), and shaken at 2,400 oscillations/min for 5 minutes. Beadbeated vials were then briefly centrifuged at 1,500 rpm for 1 second in an Eppendorf 5810R centrifuge (Eppendorf-Netheler-Hinz GmbH, Barkhausenweg, Germany). An aliquot of 200 μl of the supernatant was transferred to a ZR BashingBead Lysis Tube from ZR Soil Microbe DNA miniprep kit (Zymo Research Corporation, Irvine, CA) and bacterial DNA was extracted following manufacturer’s instructions. *Quick*-DNA™ Fungal/Bacterial Miniprep Kit (Zymo Research Corporation, Irvine, CA) was used to extract DNA from batch cultures.

### Bioinformatics approach for finding unique region for *Bacillus firmus* I-1582

To determine a fragment of the strain’s genome, which is unique relative to other strains in the *B*. *firmus* species, an in-house computational protocol was developed. This protocol essentially used NCBI’s BLAST tool (version 2.2.28+) to align whole genomes from alternate strains in *B*. *firmus* to the genome of the strain *B*. *firmus* I-1582. Genomic regions in the reference *B*. *firmus* I-1582 were then identified by fitting a profile of having at most 60% of the bases mapped to by any given alternate strain used in the analysis and meeting a minimum length requirement of 5,000 bp.

### Bioinformatics approach for finding unique region for *Bacillus amyloliquefaciens* QST713

To determine a unique region in the genome of the strain *B*. *amyloliquefaciens* QST713 which is not observed in most other *B*. *amyloliquefaciens* genomes available, a slightly different algorithm was devised. The reason for doing so was that the method used for finding a unique region in *B*. *firmus* I-1582 did not work at identifying such a region in *B*. *amyloliquefaciens* QST713. The new method devised here targeted to find the “most” unique region in *B*. *amyloliquefaciens* QST713 relative to other *B*. *amyloliquefaciens*. Similar to the other approach a binary matrix was first constructed where the rows consisted of every position in the reference genome and the columns were alternate *B*. *amyloliquefaciens* strains in our strain collection. In such a matrix, a value of 1 was used to indicate that a certain position in *B*. *amyloliquefaciens* QST713’s genome (row) was mapped upon by a given alternate strain (column) by a local alignment which had an expected value of 1e-12. A value of 0 was used to show that the position was not mapped to that alternate strain. Afterwards, the reference genome was scanned with a sliding window of size 250 bp, to increase sensitivity, and the windows with the least number of alternate *B*. *amyloliquefaciens* mapping to them were logged. These windows overlapped and a single 351 bp region was identified as being the “most” unique for the strain *B*. *amyloliquefaciens* QST713. Further searching NCBI’s nt database for the region, revealed that only 1 of 50 publicly available genomes in the *B*. *amyloliquefaciens* species, regarding strain G341, seemed to possess this region. Additionally, the region was also found in strain D2-2, which is one out of 68 *B*. *velezensis* publically available genomes,.

### qPCR assays

qPCR primers and TaqMan^®^ probes were designed using PrimerQuest software (Integrated DNA Technologies, Inc) available online (Table 1). qPCR was performed in a final volume of 20 μl in the CFX96 Real-Time PCR Detection System (Bio-Rad Laboratories Inc, Hercules, CA). Three μl of extracted DNA, as described above, was amplified with forward and reverse primers, Taqman^®^ probe (Eurofins MWG Operon LLC, Chicago, IL) and PerfeCTa Multiplex qPCR ToughMix Low ROX (Quantabio, Beverly, MA). Final concentrations of primers and probe were 250 nM and 150 nM respectively for both *B*. *firmus* I-1582 and *B*. *amyloliquefaciens* QST713. PCR amplification conditions: 95^o^ C for 15 min; 39 cycles of 95^o^ C for 15 s and 58 ^o^ C for 1 min.

**Table 1:** Primers and probes used in this study specific to *B*. *firmus* I-1582 and *B*. *amyloliquefaciens* QST713.

Primer/Probe name	Sequence (5’->3’)
Votivo_2F (forward)	CTCCAATTCCTAATATCCTGCAAAG
Votivo_2R (reverse)	GGAAAGTCACGGGACAGTTAT
Votivo_2P (probe)	TCAGGATGAGTATGCCATTCACCCA
Serenade_1F (forward)	GACGTATGGATACACCTCTTTAAT
Serenade_1R (reverse)	CCAAATTCCTCAGAAGAGAGAG
Serenade _1P (probe)	TTCCCATTAATATACTCAATTAGAGAACCT

### Quantification of bacteria

Numbers of *B*. *firmus* I-1582 or *B*. *amyloliquefaciens* QST713 bacteria associated with corn roots was quantified using standard curves constructed from spore suspensions of *B*. *firmus* I-1582 or *B*. *amyloliquefaciens* QST713 spiked with corn roots and root-associated soil, as described above. Serial dilutions of spores (10^1^ to 10^9^ cfu/ml spores) of *B*. *firmus* I-1582 or *B*. *amyloliquefaciens* QST713 were added to 5 g of corn roots with 2 mm Zirconia beads in a 7 ml plastic vial. Bacterial DNA was extracted as described above. qPCR was performed as described above and Cq values from qPCR data for each serial dilution was plotted against the log value of the number of spores in the serial dilution to get the standard curve. % efficiency was calculated by the Bio-Rad CFX Manager 3.1^™^ software (Bio-Rad Laboratories Inc, Hercules, CA) from the amplification efficiency (E) using the formula, % Efficiency = (E—1) * 100. Amplification efficiency of the qPCR reaction was calculated using the formula E = 10 ^(-1/slope)^ -1 [31]. % efficiency of 100% means perfect doubling of products with every cycle.

### Nucleotide sequence accession numbers

The GenBank accession numbers for the target DNA sequences generated here and used for strain-specific qPCR are MG581691 for strain *B*. *firmus* I-1582 and MG581692 for strain *B*. *amyloliquefaciens* QST713.

### Statistical analysis

Populations of bacteria treated with three initial inoculums in growth chambers over three weeks were compared using logistic regression analysis as bacteria counts were not normally distributed. Data analysis for greenhouse experiments and field experiments were performed by repeated measures analysis of variance (ANOVA) and two-way ANOVA respectively using SPSS software (SPSS Inc., 2016). Wild-type and antibiotic resistant strains were analyzed together when comparing both qPCR and dilution-plating. Data was normalized using natural log transformation prior to repeated measures analysis. Two-way ANOVA were conducted with normalized bacteria strain (natural log transformed), variety of corn and soybean, and their interactions as factors. Tukey’s HSD post hoc test was used to compare the means of statistically significant factors.

## Results

### Construction of standard curves for bacterial population quantification

**Fig 1:**
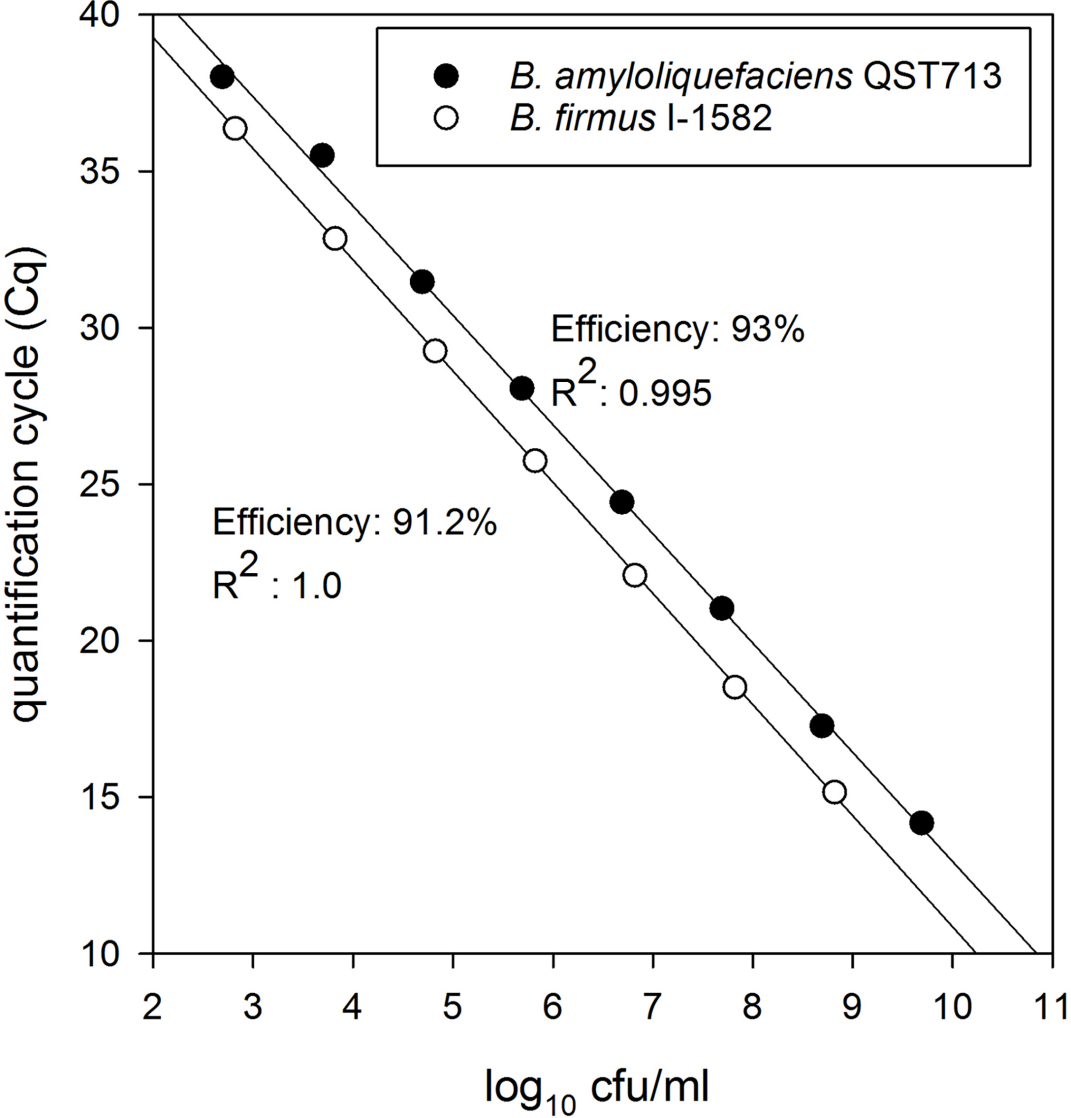
qPCR standard curves of *B*. *firmus* I-1582 and *B*. *amyloliquefaciens* QST713. Standard curves were plotted from Cq values obtained by qPCR of spiked spore suspensions of *B*. *firmus* I-1582 and *B*. *amyloliquefaciens* QST713. Spiked spore suspensions were prepared from adding 5 g of corn roots and associated soil into 2 ml of spore suspensions with known CFU.

Two standard curves were constructed to quantify *B*. *firmus* I-1582 and *B*. *amyloliquefaciens* QST713 cells associated with corn and soybean roots. A *B*. *firmus* I-1582 spore suspension of 6.6 x 10^8^ cfu/ml was used to construct *B*. *firmus* I-1582 standard curve. Seven serial dilutions ranging from 6.6 x 10^8^ to 6.6 x 10^2^ cfu/ml, covering 6 orders of magnitude was used in the standard curve. The efficiency was 91.2% and the coefficient of correlation (R^2^) was 1.000 for *B*. *firmus* I-1582 standard curve (Fig 1). Similarly, a *B*. *amyloliquefaciens* QST713 spore suspension of 4.9 x 10^9^ cfu/ml was used to construct *B*. *amyloliquefaciens* QST713 standard curve. Eight serial dilutions ranging from 4.9 x 10^9^ to 4.9 x 10^2^ cfu/ml, covering 7 orders of magnitude was used in the standard curve. The efficiency was 93.0% and the coefficient of correlation (R^2^) was 0.995 for *B*. *amyloliquefaciens* QST713 standard curve (Fig 1).

### Quantification of specific bacterial strains in corn roots in growth chamber assays

**Fig 2:**
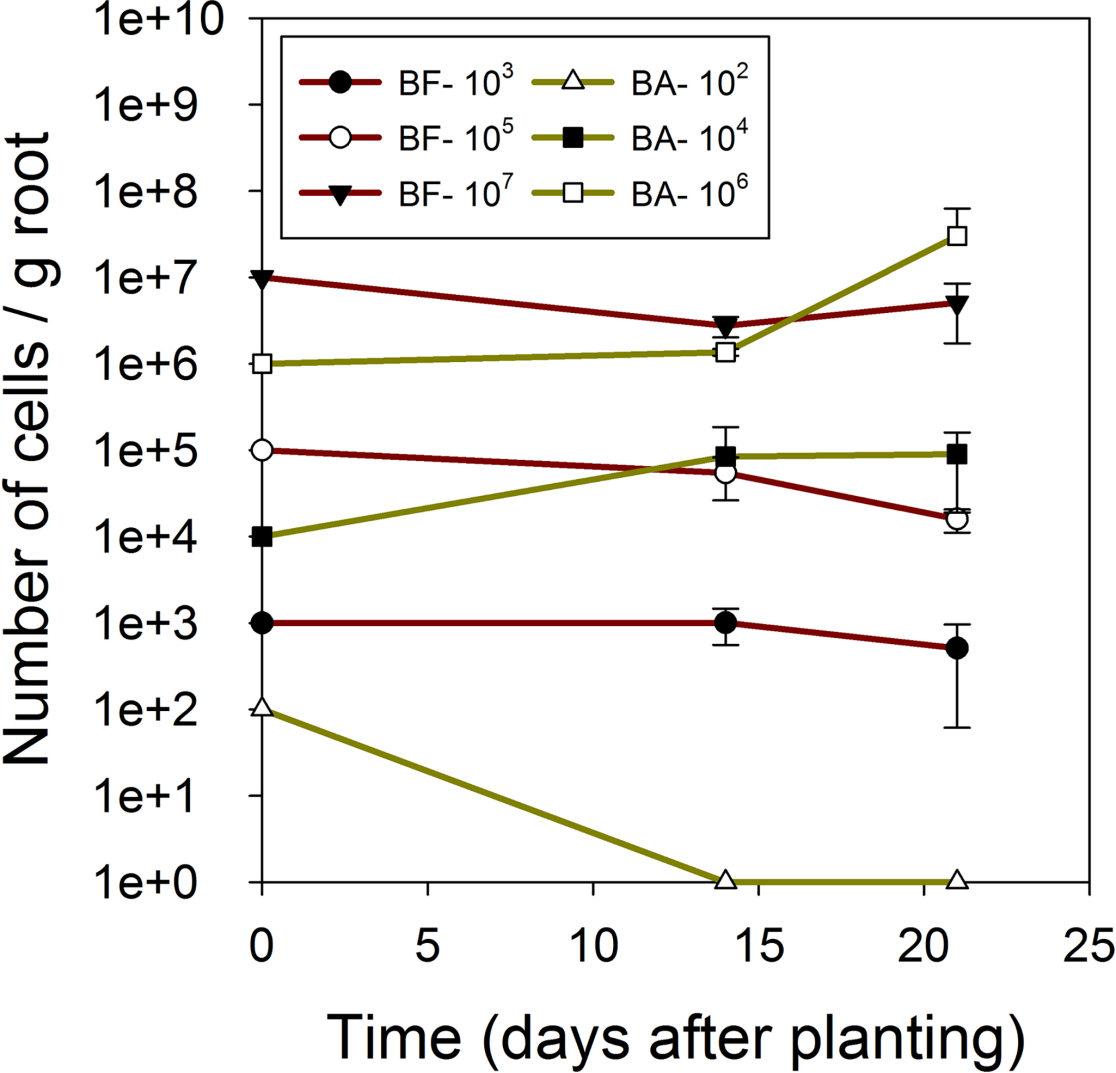
Quantification of root associated bacteria in corn seedlings grown under sterile conditions in growth chambers. Corn seeds were treated with 10^7^ (BF-10^7^), 10^5^ (BF-10^5^), or 10^3^ (BF-10^3^) spores of *B*. *firmus* I-1582; and 10^6^ (BA-10^6^), 10^4^ (BA-10^4^), or 10^2^ (BA-10^2^) spores of *B*. *amyloliquefaciens* QST713 per seed. Root sampling was carried out at 14 and 21 days after planting (n = 2). Number of cells at t = 0 days was estimated based on the initial seed treatment. ND = non-detectable.

As shown in Fig 2, bacterial populations were detected in most cases in corn roots for both *B*. *firmus* I-1582 and *B*. *amyloliquefaciens* QST713. Populations at time = 0 were calculated based on initial inoculum applied to seeds. Both strains showed continuous root colonization up to 3 weeks after planting for all treatments. No significant differences (*P* = 0.318) were observed for populations of bacteria over time considering each initial inoculum and strain separately at day 14 and day 21. The exception was the treatment with 10^2^ spores of *B*. *amyloliquefaciens* QST713 (BA-10^2^), that was not detected in corn seedlings at days 14 and 21 (Fig 2). This may be due to the presence of lower number of bacteria on the roots below the detection limit of this assay.

### Quantification of wild-type and antibiotic-resistant strains in corn roots in greenhouse assays

**Fig 3:**
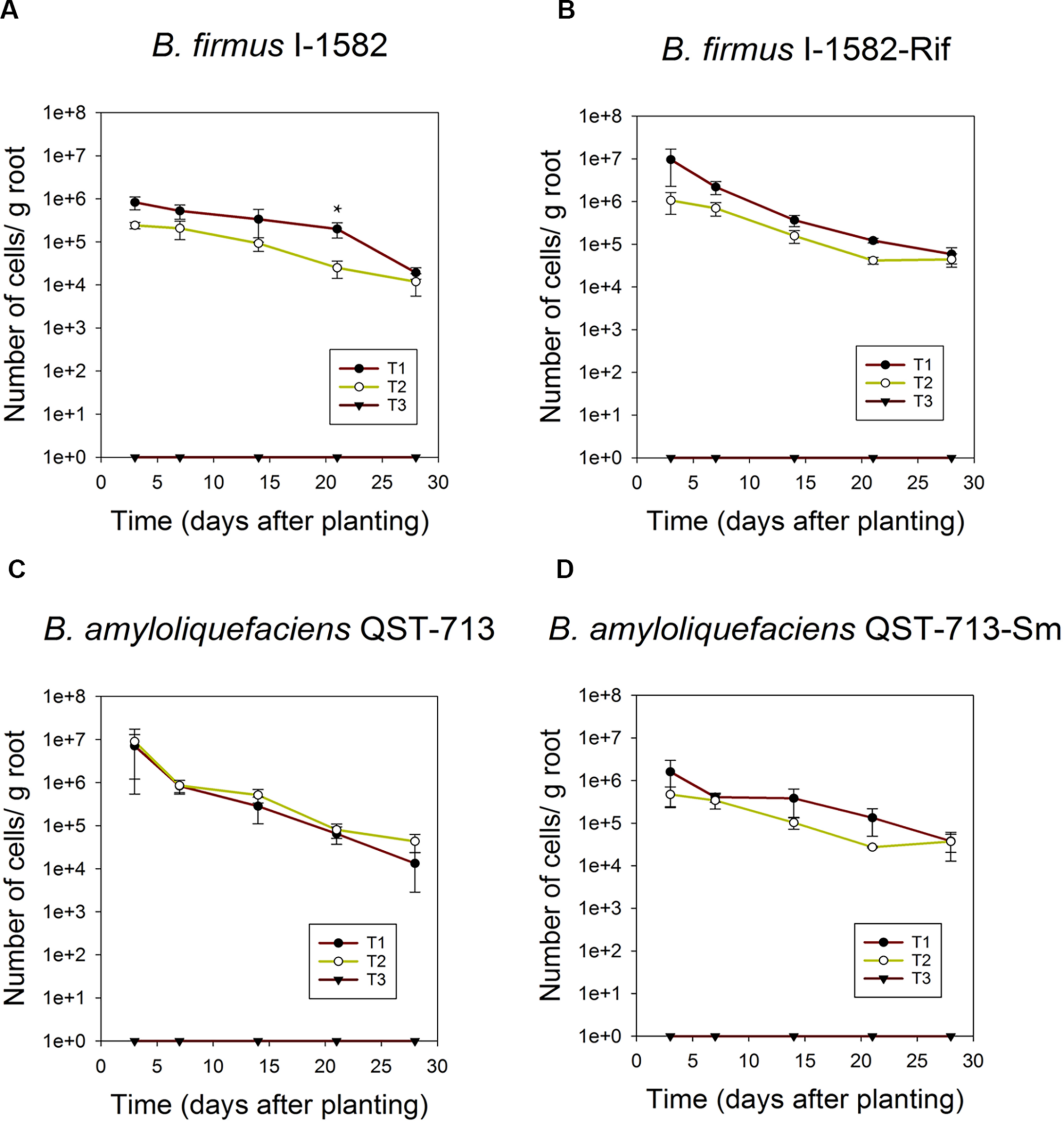
Quantification of root associated bacteria in corn seedlings grown in natural soil in the greenhouse. Corn seeds were treated on each case with: **(A)** 10^7^ spores of *B*. *firmus* I-1582 alone (T1), mixed with 10^7^ spores of *B*. *amyloliquefaciens* QST713 (T2) or non-treated (T3); **(B)** 10^7^ spores of *B*. *firmus* I-1582-Rif alone (T1), mixed with 10^7^ spores of *B*. *amyloliquefaciens* QST713-Sm (T2), or non-treated (T3); **(C)** 10^7^ spores of wild-type *B*. *amyloliquefaciens* QST713 alone (T1), mixed with 10^7^ spores of *B*. *firmus* I-1582 (T2), or non-treated (T3). **(D)** 10^7^ spores of *B*. *amyloliquefaciens* QST713-Sm alone (T1), mixed with 10^7^ spores of *B*. *firmus* I-1582-Rif (T2), or non-treated (T3). In **(A)** and **(B)** *B*. *firmus* I-1582 specific primers and probe were used for qPCR bacterial population determination; while in **(C)** and **(D)** *B*. *amyloliquefaciens* QST713 specific primers and probe were used for qPCR (n = 3 seedlings/time point and error bars show SE). Repeated measures analysis of variance was performed at each data point comparing T1 and T2, Tukey’s HSD post hoc test was used to compare the means of statistically significant factors. Symbol * indicates time points that do not overlap 95% confidence intervals between T1 and T2 treatments.

As shown in Fig 3, *B*. *amyloliquefaciens* QST713 and *B*. *firmus* I-1582 wild-type and antibiotic resistant strains associated with corn roots were detectable up to 4 weeks using the qPCR protocol. Presence of indigenous microflora in soil or inoculation with another PGPR strain did not interfere with quantification. When populations of *B*. *firmus* I-1582 were quantified with specific primers, there was no significant difference (*P* = 0.548) in the number of wild-type *B*. *firmus* I-1582 bacteria inoculated alone or 1:1 mixture with *B*. *amyloliquefaciens* QST713 at all data points. We observed that *B*. *firmus* I-1582 bacteria inoculated alone had significantly higher *B*. *firmus* I-1582 bacteria population compared to 1:1 mixture with *B*. *amyloliquefaciens* QST713 at day 21 based on the 95% confidence intervals at that particular time point (Fig 3A). There was no significant difference (*P* = 0.548) in number of *B*. *firmus* I-1582-Rif bacteria detected in corn seedlings inoculated with this strain alone or as a mixture (1:1) with *B*. *amyloliquefaciens* QST713-Sm at all data points (Fig 3B).

There was no significant difference (*P* = 0.208) in number of wild-type *B*. *amyloliquefaciens* QST713 bacteria detected in corn seedlings inoculated alone or as a 1:1 mixture with wild-type *B*. *firmus* I-1582 (Fig 3C). Similar results were found when antibiotic-resistant strains were used. There was no significant difference (*P* = 0.208) in number of *B*. *amyloliquefaciens* QST713-Sm bacteria detected in corn seedlings inoculated alone or as a 1:1 mixture with *B*. *firmus* I-1582-Rif (Fig 3D).

**Fig 4:**
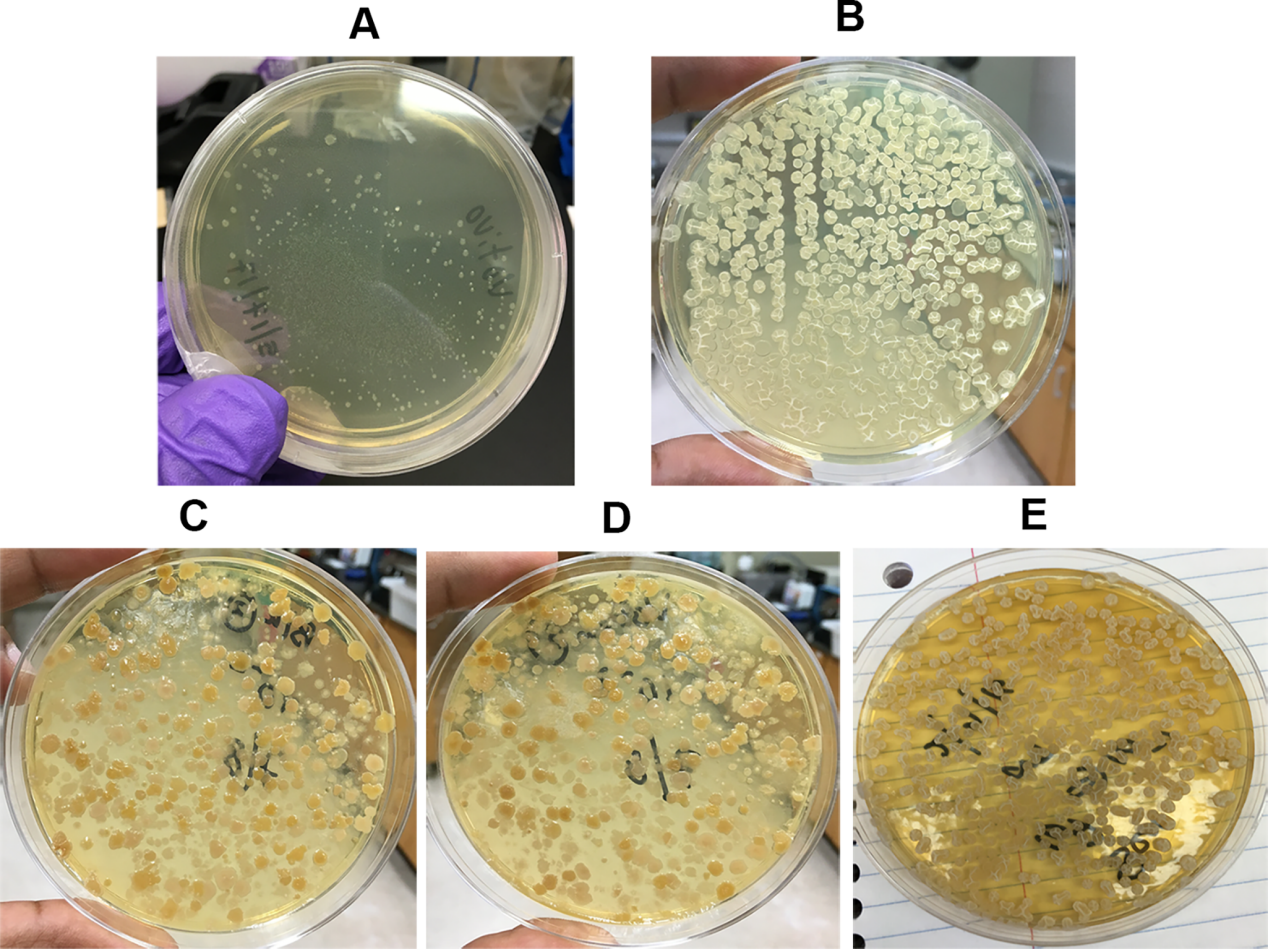
Comparison of bacteria colony morphology on TSA plates. **(A)** *B*. *firmus* I-1582 colonies from pure culture **(B)** *B*. *amyloliquefaciens* QST713 colonies from pure culture **(C)** Rhizosphere bacteria from untreated corn seedlings **(D)** Rhizosphere bacteria from *B*. *firmus* I-1582 treated corn seedlings **(E)** Rhizosphere bacteria from *B*. *amyloliquefaciens* QST713 treated corn seedlings.

Comparing our qPCR protocol with dilution-plating of wild-type strains was investigated, but it was difficult to distinguish *B*. *firmus* I-1582 or *B*. *amyloliquefaciens* QST713 colonies from other soil bacteria colonies in TSA plates (Fig 4). Therefore, we decided to use antibiotic-resistant strains for comparison, since population dynamics of wild-type and antibiotic-resistant derivatives were similar (Fig 3A vs. 3B, 3C vs. 3D).

**Fig 5:**
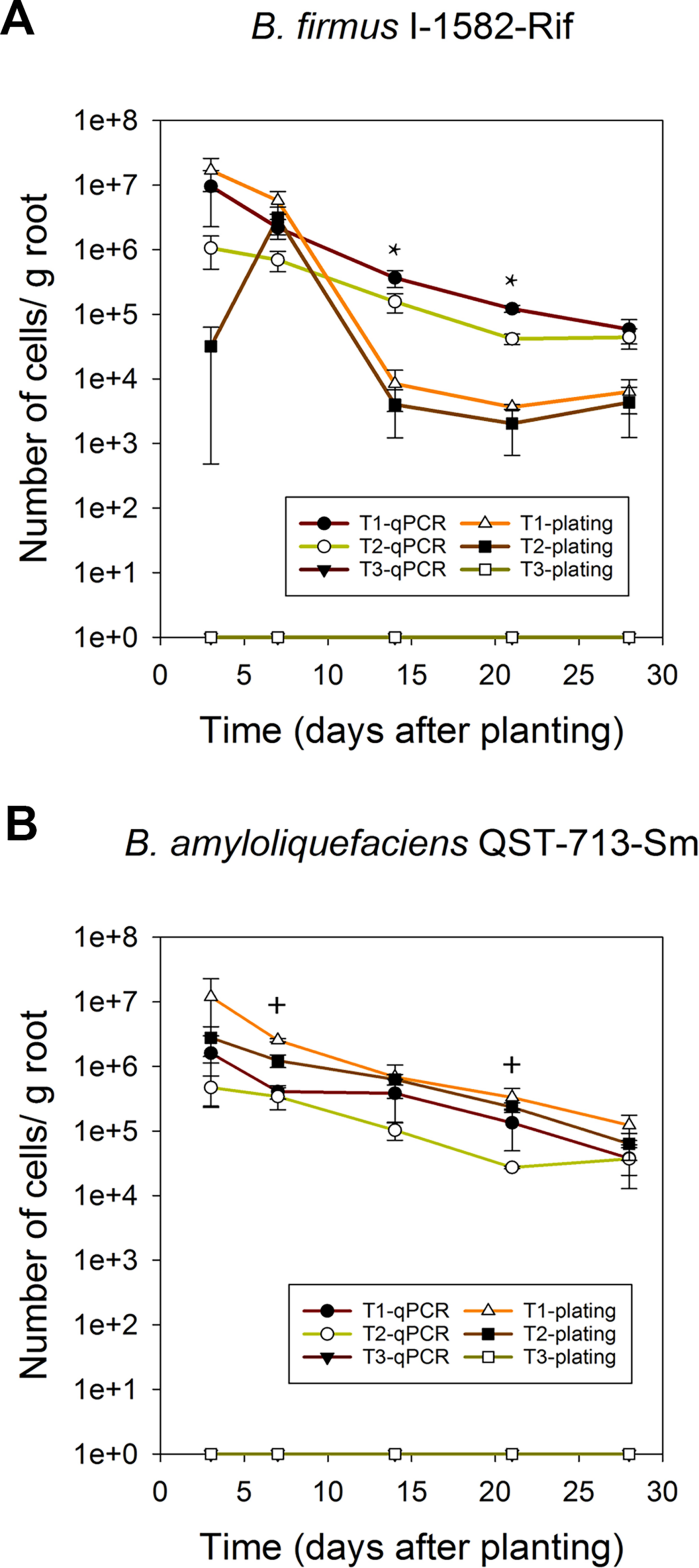
Comparison of bacteria root colonization of greenhouse grown corn seedlings by qPCR and dilution-plating. **(A),** *B*. *firmus* I-1582-Rif and **(B),** *B*. *amyloliquefaciens* QST713-Sm were quantified by qPCR and dilution-plating on TSA_Rif_ and TSA_Sm_ plates, respectively. Corn seeds were treated with 10^7^ spores of each strain inoculated alone (T1), 10^7^ spores of each strain mixed together (T2) or non-treated (T3) as described in the methods. Values represent means and error bars show standard error (n = 3 seedlings/time point). Repeated measures analysis of variance was performed separately for T1 and T2 for each data point comparing qPCR and plating measurements. Symbol * indicates time points that do not overlap 95% confidence intervals between qPCR and plating for both T1 and T2 treatments. Symbol + indicates time points that do not overlap 95% confidence intervals between qPCR quantification and dilution-plating only for T2.

Numbers of bacteria associated with corn roots were estimated for *B*. *firmus* I-1582-Rif and *B*. *amyloliquefaciens* QST713-Sm strains by qPCR and dilution-plating to compare the two techniques (Fig 5A and 5B, respectively). There was no significant difference (*P* = 0.138) in the number of *B*. *firmus* I-1582-Rif bacteria estimated from qPCR quantification and dilution-plating at all the data points. We observed that qPCR quantification estimated a significantly higher number of *B*. *firmus* I-1582 compared to dilution-plating at day 14, and day 21 in corn seedlings treated with *B*. *firmus* I-1582-Rif alone and as a mixture (1:1) with *B*. *amyloliquefaciens* QST713-Sm based on the 95% confidence intervals at that particular time points (T1 and T2, Fig 5A).

There were no significant difference (*P* = 1.852) in the numbers of *B*. *amyloliquefaciens* QST713-Sm bacteria estimated from qPCR quantification and dilution-plating at all data points (day 3, day 7, day 14, day 21 and day 38) when corn seedlings were treated with *B*. *amyloliquefaciens* QST713-Sm alone (T1). Similarly, there were no significant differences (*P* = 0.852) in the numbers of *B*. *amyloliquefaciens* QST713-Sm bacteria estimated from qPCR quantification and dilution-plating at all data points when bacteria was inoculated as part of a mixture (1:1) of *B*. *amyloliquefaciens* QST713-Sm and *B*. *firmus* I-1582-Rif (T2). We observed that dilution-plating estimated a significantly higher number of *B*. *amyloliquefaciens* QST713-Sm compared to qPCR quantification at day 7 and day 21 in corn seedlings treated as a mixture (1:1) of *B*. *amyloliquefaciens* QST713-Sm and *B*. *firmus* I-1582-Rif (T2) based on the 95% confidence intervals at that particular time points (Fig 5B).

### Quantification of specific bacterial strains in corn and soybean in US field trials of Crop Science Division, Bayer

**Fig 6:**
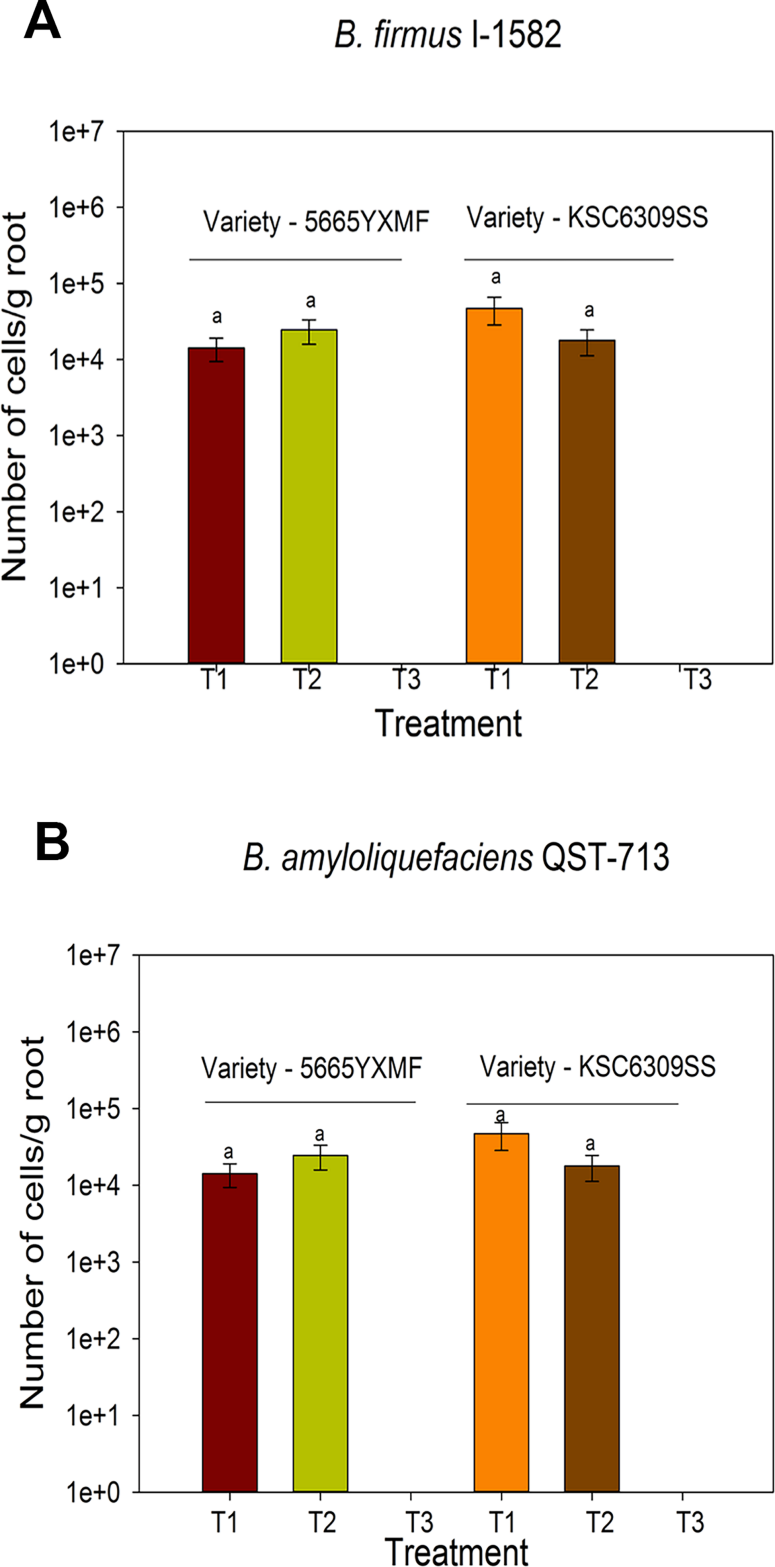
Root colonization of field grown corn seedlings by *B*. *firmus* I-1582 and *B*. *amyloliquefaciens* QST713. Corn seeds were treated with **(A)** 10^6^ spores of *B*. *firmus* I-1582 (T1 and T2) and **(B)** 10^6^ spores of *B*. *amyloliquefaciens* QST713 (T1 and T2). T3 = uninoculated. qPCR quantification was performed with *B*. *firmus* I-1582 or *B*. *amyloliquefaciens* QST713 specific primers and probe using a standard curve. Error bars show Standard Error (n = 5 seedlings). Same letters on top of bars represent non-significant differences (*P* = 0.387) for each variety according to two-way ANOVA.

**Fig 7:**
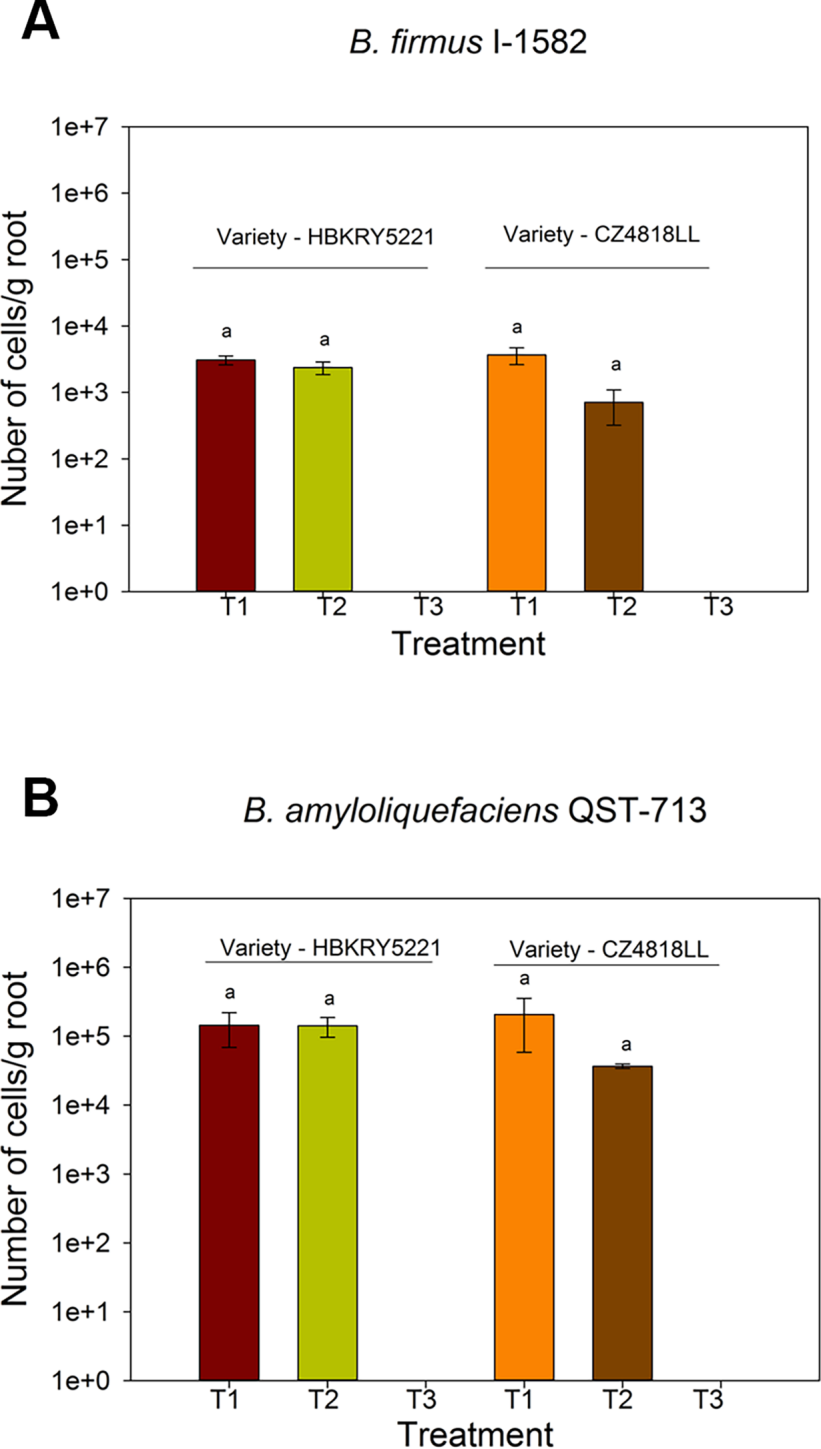
Root colonization of field grown soybean seedlings *B*. *firmus* I-1582 and *B*. *amyloliquefaciens* QST713. Soybean seeds were treated with **(A)** 10^6^ spores of *B*. *firmus* I-1582 (T1 and T2) and **(B)** 10^6^ spores of *B*. *amyloliquefaciens* QST713 (T1 and T2). T3 = uninoculated. qPCR quantification was performed with *B*. *firmus* I-1582 or *B*. *amyloliquefaciens* QST713 specific primers and probes using a standard curve. Error bars show Standard Error. (n = 5 seedlings). Same letters on top of bars represent non-significant differences (*P* = 0.665) for each variety according to two-way ANOVA.

Root colonization of soybean and corn by wild-type *B*. *amyloliquefaciens* QST713 and *B*. *firmus* I-1582 was quantified in the field. There was no significant difference (*P* = 0.387) in the number of *B*. *firmus* I-1582 or *B*. *amyloliquefaciens* QST713 bacteria associated with two corn varieties tested in field trials (Fig 6A and 6B). Similarly, there was no significant difference (*P* = 0.665) in the number of *B*. *firmus* I-1582 or *B*. *amyloliquefaciens* QST713 bacteria associated with two soybean varieties (Fig 7A and 7B). According to these results, both *B*. *firmus* I-1582 and *B*. *amyloliquefaciens* QST713 do not show a significant variety dependent or plot level differences in corn and soybean root colonization. Fig 6 and Fig 7 confirm that our qPCR protocol can be successfully applied to quantitate root colonization by PGPR in multiple crops grown in the field. In addition, *B*. *firmus* I-1582-treated corn and soybean seedlings were analyzed with *B*. *amyloliquefaciens* QST713 specific primers; and *B*. *amyloliquefaciens* QST713-treated corn and soybean seedlings with *B*. *firmus* I-1582 specific primers, to see if there was any cross-reaction or contamination. According to qPCR results, there was no cross-reaction or contamination in *B*. *firmus* I-1582 treated corn and soybean seedlings and *B*. *amyloliquefaciens* QST713 treated corn and soybean seedlings.

## Discussion

The aim of this study was to develop a qPCR-based protocol to quantitate total root colonization by two PGPR strains, *B*. *firmus* I-1582 and *B*. *amyloliquefaciens* QST713 in corn seedlings grown in natural soil. Quantification of root colonization by PGPR is an essential part of tracking inoculants in the field, allowing studies of persistence and understanding of environmental fate of introduced microbes applied in the soil, as well as discovering and evaluating potential PGPR strains. With more interest in preserving soil health and exploiting soil microbiome, there are more microbe-based seed and soil products being made available to the growers, therefore differentiating strains and products is fundamental for the evaluation of the environmental impact of these inoculants. Despite several microscopy and PCR based quantification methods described recently and in the past, dilution-plate counting is still the most common method used in quantification of root colonization by PGPR bacteria in greenhouse and in field under non-sterile soil. One of the common drawbacks of PCR based methods is that these methods are not applicable to non-sterile conditions in greenhouse or in field [27]. However, dilution-plate counting is laborious, and sample processing and plating has to be carried out immediately after sampling. Therefore, a faster and more convenient quantification method to quantitate root and rhizosphere colonization of PGPR is required. The method we describe here to quantitate root colonization of PGPR can successfully replace dilution-plate counting method and has the advantage of being strain-specific.

This is the first comprehensive report describing a strain-specific qPCR based protocol to quantitate PGPR strains associated with crops grown in non-sterile soil and under field conditions. Previously described PCR based quantification protocols were not strain-specific or required laborious processing such as root washing step to minimize interference from natural microflora associated with roots or bacteria enrichment steps [32–35]. Root washing could potentially lead to a loss of root-associated bacteria of interest together with native microflora. With strain-specific primers and probes, we were able to bypass the requirement of root washing step. The method of recovering inoculated bacteria from roots is an important part of any quantification technique. Presumably, soil particles loosely attached to roots may contain several log units higher of the inoculated bacteria than in the rhizosphere soil without roots [1]. Therefore, we used a gentle root shaking in our protocol to remove loosely attached soil particles to provide a more accurate estimate of root colonization. However, increasing soil mass attached to roots does not significantly increase estimation of inoculated bacteria population densities [36]. Therefore, gentle shaking of roots would not lead to a significant loss of bacteria of interest and result in lower qPCR detection limit.

Couillerot and Bouffaud et al. described an *Azospirillum lipoferum* CRT1 strain-specific qPCR based protocol [37]. Their protocol required laborious sample processing such as lyophilization and the detection limit was 4 x 10^4^ cells per g, which is a higher detection limit compared to protocol described here. Therefore, they were only able to quantitate *A*. *lipoferum* CRT1 in maize rhizosphere up to 10 days in non-sterile soil. Pereira et al. have recently described a *Herbaspirillum seropedicae* SmR1 strain-specific qPCR protocol to quantitate root colonization of maize in sterile conditions. However they were only able to detect *H*. *seropedicae* SmR1 in maize roots in non-sterile soil and did not perform any quantification of bacteria [38]. Von Felten et al described a qPCR protocol to detect 3 *Pseudomonas fluorescens* strains in maize rhizosphere. Their protocol requires a 24-hour lyophilization step before DNA extraction from maize roots and associated soil [39]. Mosimann et al also described a qPCR protocol to detect 2 *Pseudomonas* strains [40]. They have used 3 different potting mixes instead of natural soil to plant maize seedlings. Potting soil could be much more homogenious and it could contain far less native microflora compared to natural soil [39] However, both Von Felten et al and Mosimann have not shown that their qPCR protocols could successfully analyze and quantitate respective PGPR strains in natural soils in field conditions.

Previous studies have compared strain-specific qPCR protocols with dilution-plating [37]. However, these studies were carried out in sterile soil. Competition and interference from native microflora could affect root colonization pattern of PGPR as well as detection limits of the qPCR protocol. Therefore, we compared our qPCR results with dilution-plating with *B*. *firmus* I-1582-Rif and *B*. *amyloliquefaciens* QST713-Sm strains (Fig 5). Estimation of root colonization by dilution-plating could underestimate the actual value if some of the cells fail to develop into colonies. We did not observe a significantly different (*P* = 0.138) estimation of root colonization of *B*. *firmus* I-1582-Rif with dilution-plating compared to that of qPCR estimation for three out of five root samplings days up to 28 days when corn seedlings were treated with *B*. *firmus* I-1582-Rif alone or as a mixture (1:1) with *B*. *amyloliquefaciens* QST713-Sm. There was no significant difference (*P* = 0.852) in estimation of root colonization of *B*. *amyloliquefaciens* QST713-Sm with dilution-plating compared to that of qPCR estimation for three out of five root samplings up to 28 days when corn seedlings were treated with a mixture (1:1) comprising*B*. *amyloliquefaciens* QST713-Sm and *B*. *firmus* I-1582-Rif. Furthermore, we did not observe a significant difference (*P* = 0.852) in estimation of *B*. *amyloliquefaciens* QST713-Sm in qPCR quantification compared to dilution-plating at all time root samplings when corn seedlings were treated with *B*. *amyloliquefaciens* QST713-Sm alone. However, there were few data points where qPCR quantification resulted in significantly higher estimation of *B*. *firmus* I-1582-Rif compared to that of dilution-plating when considered 95% confidence intervals (Fig 5A). High qPCR estimation of *B*. *firmus* I-1582-Rif could result from amplification of DNA from dead cells, lack of germination of spores in media used, or the possibility of a state similar to viable but non culturable. Although less frequent than high qPCR estimation compared to dilution-plating, there were two incidences out of ten pairwise comparisons where dilution-plating resulted in higher estimation of *B*. *amyloliquefaciens* QST713-Sm compared to that of qPCR quantification, only in cases where this strain was co-inoculated with *B*. *firmus* I-1582-Rif (T2, Fig 5B). A possible explanation could be that the reduction in population of *B*. *amyloliquefaciens* QST713-Sm is only detectable by qPCR in soils, but when growing alone in plates due to antibiotic selection, without competing with *B*. *firmus* I-1582-Rif, that reduction in population is lost These aspects will be studied in future research aimed to elucidate the basis for these differences.

We were able detect as low as 10^3^ CFU/g using our qPCR protocol (Figs 1 and 2). Even though we investigated this protocol extensively in corn, results obtained from field-grown soybean show that this protocol could be applied to investigate root colonization of the same bacteria strains in other crop species without any modifications (Fig 7). Use of this method is advantageous for large field trials, as this protocol requires minimal processing prior to long-term storage of roots at -80^o^ C. Furthermore, we did not observe any cross-reaction of primer pairs or cross-contamination of the two strains used in this study. Therefore, our protocol is suitable to investigate interactions of two or more PGPR strains in a single crop.

We obtained whole root systems during sample processing whenever possible as it would give maximum amount of information about root colonization [1]. We had to obtain representative segments of root system during latter stages of the greenhouse experiments since root mass was higher than 5 g. We have expressed root colonization of *B*. *firmus* I-1582 and *B*. *amyloliquefaciens* QST713 strains in corn and soybean roots as number of cells per gram of roots since we were interested in quantifying total root colonization. More soil particles could be attached to roots if the soil is moist. That could result in underestimation of bacteria populations since the root surface has higher bacterial population densities than adjacent soil [1,41]. We avoided excessive soil moisture during root sampling and selected approximately 5 g of roots whenever possible to prevent underestimation of root-associated bacteria.

According to our results, we were able to quantitate root colonization of *B*. *amyloliquefaciens* QST713 and *B*. *firmus* I-1582 in corn seedlings grown in greenhouse and in field in non-sterile soil. Using dilution-plating of *B*. *amyloliquefaciens* QST713-Sm and *B*. *firmus* I-1582-Rif strains, we were able to show that our qPCR quantification was accurate. Furthermore, this protocol can be successfully extended to quantification of other bacteria strains in other crops. Our qPCR protocol is also suitable to quantitate both total root colonization as well as internal and external root colonization, provided the number of bacteria falls within detection limits of our qPCR protocol.

We have studied corn root colonization of *B*. *firmus* I-1582 and *B*. *amyloliquefaciens* QST713 strains over 4 weeks in non-sterile soil. We observed a steady root colonization in corn roots by both *B*. *firmus* I-1582 and *B*. *amyloliquefaciens* QST713 strains over 28 days. We have also included mixed inoculations of *B*. *firmus* I-1582 and *B*. *amyloliquefaciens* QST713 together. Presence of *B*. *firmus* I-1582 or *B*. *amyloliquefaciens* QST713 in the rhizosphere did not affect the final population size of either *B*. *amyloliquefaciens* QST713 or *B*. *firmus* I-1582 at day 28. We have not investigated these two strains together in a single crop in field. Further investigation of mixed inoculations of *B*. *firmus* I-1582 and *B*. *amyloliquefaciens* QST713 in field would provide more information on each strains root colonization pattern regarding any distinct niches in the corn rhizosphere or if there are any antagonistic effects between two strains.
